# Comparative RNA-seq analysis reveals a critical role for ethylene in rose (*Rosa hybrida*) susceptible response to *Podosphera pannosa*

**DOI:** 10.3389/fpls.2022.1018427

**Published:** 2022-09-27

**Authors:** Xintong Liu, Peihong Fang, Zicheng Wang, Xiaoqian Cao, Zhiyi Yu, Xi Chen, Zhao Zhang

**Affiliations:** ^1^ Beijing Key Laboratory of Development and Quality Control of Ornamental Crops, Department of Ornamental Horticulture, College of Horticulture, China Agricultural University, Beijing, China; ^2^ School of Agronomy and Horticulture, Jiangsu Vocational College of Agriculture and Forest, Jurong, China; ^3^ Engineering and Technical Center for Modern Horticulture, Jurong, China; ^4^ Horticulture College, Hainan University, Haikou, China

**Keywords:** powdery mildew, transcriptome, transcription factors, rose leaf, 1-MCP

## Abstract

Rose is one of the most important ornamental flowers, accounting for approximately one-third of the world’s cut flower market. Powdery mildew caused by *Podosphera pannosa* is a devastating fungal disease in rose, mainly infecting the young leaves and causing serious economic losses. Therefore, a study on the mechanism of the fungus infecting the rose leaves and the possibility to improve resistance hereby is interesting and meaningful. Accordingly, we conducted transcriptome sequencing of rose leaves infected by *P. pannosa* at different time points to reveal the molecular mechanism of resistance to powdery mildew. The high-quality reads were aligned to the reference genome of *Rosa chinensis*, yielding 51,230 transcripts. A total of 1,181 differentially expressed genes (DEGs) were identified in leaves during *P. pannosa* infection at 12, 24, and 48 hpi. The transcription factors of ERF, MYB, bHLH, WRKY, *etc*., family were identified among DEGs, and most of them were downregulated during *P. pannosa* infection. The Kyoto Encyclopedia of Genes and Genomes analysis showed that the hormone signal transduction pathway, especially ethylene signal-related genes, was consistently showing a downregulated expression during powdery mildew infection. More importantly, exogenous 1-MCP (inhibitor of ethylene) treatment could improve the rose leaves’ resistance to *P. pannosa*. In summary, our transcriptome of rose leaf infected by powdery mildew gives universal insights into the complex gene regulatory networks mediating the rose leaf response to *P. pannosa*, further demonstrating the positive role of 1-MCP in resistance to biotrophic pathogens.

## Introduction

*Rosa hybrida* (modern rose) is independently domesticated in both Europe and China several thousands of years ago (Rusanov et al., 2009). Rose is one of the most important cut flowers and ornamental plants worldwide. Its planting area and market size rank as the first in global flower production, which means a remarkable commercial value. However, the rose is vulnerable to fungal diseases during cultivation and post-harvest transport, which cause huge economic losses. Among them, powdery mildew (PM) is one of the most common but devastating fungal diseases in the world (Debener and Byrne, 2014). In addition to the diseases of rose, it also affects other *Roseceae* plants, like apple, strawberry, pear, *etc*., and it is more serious in the greenhouse than in the open field. There are more than 650 species of *Erysiphales* in Ascomycota, including 15 sexual and four asexual species. This fungus has a wide range of hosts. More than 650 species of monocotyledonous plants and over 9,000 species of dicotyledonous plants have been reported (Schulzelefert and Vogel, 2000).

Rose powdery mildew was caused by *Podospheara pannosa* (*Pp*.). The fungus is a biotrophic ascomycete. In a warm, dry, or humid environment, PM infects the green vegetative organs such as young leaves and shoots of rose (Debener and Byrne, 2014). The rapid spread of PM causes the withered leaves of the plants to shrivel, shrink, or even fall off, and the surface is covered with white powder. PM is greatly damaging the ornamental and economic value of rose worldwide. An effective way to control the disease is to improve the resistance of plants (Vielba-Fernández et al., 2020). Comparative transcriptional analysis, using RNA-based sequencing (RNA-seq), is a common strategy of understanding the molecular mechanism of plant–pathogen interactions. This method has been widely applied in research about plant–pathogen interactions in horticultural crops, including rose (*Rosa hybrida*) (Liu et al., 2018), grape (*Vitis vinifera*) (Toffolatti et al., 2018), and apple (*Malus × domestica*) (Tian et al., 2019). However, to date, the lack of transcriptomic data on rose–PM interactions, coupled with the limitations of transgenesis and gene editing on rose, led to breeding in rose against PM with little progress achieved.

Normally, the onset of plant resistance to pathogens begins from the recognition of pathogens by plants, followed by the cascade of transmission of disease resistance signals depending on PAMP-triggered immunity and effector-triggered immunity. Signaling molecules and transcription factors (TFs) are the key components in both basal and race-specific immunity (Yuan et al., 2021). Plant hormones SA, JA, and ethylene (ET) are considered to be the main regulatory signals related to plant disease resistance. It is generally believed that SA is mainly the downstream signal of defense responding to the biotrophic fungi. JA and ET are involved in the downstream signal pathway of the defense response of necrotrophic fungi (Glazebrook, 2005). Studies have shown that, after a biotrophic infection, the plant cells in the infected area would accumulate SA in a short time (Duan et al., 2015). The function of plant defense-related TFs has also been reported in recent years. Overexpression of *VvZIP60* enhanced grape resistance to PM *via* the SA signaling pathway (Yu et al., 2019). Meanwhile, overexoression of *CmbHLH87* in pumpkin (Guo et al., 2020), HvNAC6 in barley (Chen et al., 2013), and FvWRKY42 (Wei et al., 2018) in strawberry enhanced the resistance to PM as a whole. However, a systematic study of the genes involved in rose early resistance to PM is still lacking.

Here we investigated the transcriptome dynamics of rose seedlings following *Pp.* primary, secondary, and tertiary infection, expecting to dig out more genes related to the resistance of rose to PM. Our aim is to provide a relevant rose gene pool with resistance to powdery mildew in order to improve the quality and the technology foundation, thereby achieving rose varieties resistant to powdery mildew and improving the commodity value of rose. Meanwhile, it is of great theoretical and practical significance to improve the plant disease resistance of powdery mildew by genetic engineering.

## Materials and methods

### Plant and fungal growth

*Rosa hybrida* ‘Samantha’ was propagated by tissue culture (Fang et al., 2021). Rose shoots with at least two leaves were cultured on half-strength Murashige and Skoog (MS) medium supplemented with 0.1 mg L^-1^ α-naphthaleneacetic (NAA) for 30 days at 22°C under a 16-h/8-h light/dark photoperiod for rooting. The rooted plants were transferred to pots containing peat moss/vermiculite (1:1) and grown at 25/16°C, 16-h/8-h light/dark photoperiod, and 60% humidity.

The powdery mildew *Podosphaera pannosa*, *Pp*, strain was cultivated with ‘Samantha’ seedlings at 25/16°C and 16-h/8-h light/dark photoperiod. Spore inoculums were prepared by harvesting spores from rose leaves in deionized water. The impurities were removed by centrifugation at 5,000 rmp for 10 min, and the culture was resuspended in distilled water to achieve experimental concentration.

### Plant infection

For the RNA-seq samples, the healthy rose seedlings with young leaves (after having been transferred to pots for 4 weeks) are evenly inoculated with powdery mildew spore suspension with a concentration of 5 × 10^5^/ml by spraying method to ensure that each leaf can be inoculated with powdery mildew. All the seedlings were covered with white film to ensure nearly 100% humidity. The seedlings without roots were harvested at 12, 24, and 48 hours post-inoculation (hpi), immediately frozen in liquid nitrogen, and stored at -80°C for further use.

For the leaf discs, young stems with three to five pairs of dark red pinnate compound leaves were cut from ‘Samantha’ grown in glasshouses in Nankou, Changping District, Beijing, China, and their stems were immediately placed in water. Rose leaves, after the hormone treatments, were punched into 12.5-mm disks and placed in 0.4% water agar with back-up, at 16 disks per petri dish. Then, each leaf disc was sprayed with 1 × 10^7^/ml powdery mildew spore suspension. The leaf discs were dried at room temperature before the petri dishes were closed. After 48 h of dark treatment, these were switched to a light culture grown at 25/16°C and 16-h/8-h light/dark photoperiod. The lesion sizes were measured at 6, 9, 12, and 15 dpi and analyzed statistically by Student’s *t*-test.

### Total RNA extraction and RNA-seq library preparation

Total RNA was extracted using the hot borate method as previously described (Wu et al., 2017), and the cultures were treated with RNase-free DNase I (Promega) to remove any contaminating genomic DNA. Two biological repeats were performed for each time point after the infection. Strand-specific RNA libraries were constructed using a protocol described previously (Jiang et al., 2011) and sequenced on the HiSeq2500 system according to the manufacturer’s instructions. The raw reads were deposited in the NCBI SRA database under accession no. PRJNA661227.

### RNA-seq data analysis

Firstly, the raw data was cleaned by removing the adaptor-containing sequences, the poly-N adaptor-containing sequences, and the low-quality reads (-L = 20, -*q* = 0.5). The clean reads were evaluated by FastQC (Brown et al., 2017). The reference genome of *Rosa chinensis* ‘Old blash’ (RchiOBHm-V2, GCF_002994745.1) was downloaded from the website (https://lipm-browsers.toulouse.inra.fr/pub/RchiOBHm-V2/) (Raymond et al., 2018). The reference genome index was constructed with Bowtie v2.2.3, and the reads were aligned to the reference genome with TopHat v2.0.12. All genes were annotated by non-redundant protein (NR), NCBI non-redundant transcript (NT), and Kyoto Encyclopedia of Genes and Genomes (KEGG) and Gene Ontology (GO) libraries with *e*-value ≤10^-5^.

The fragments per kilobase per million reads (FPKM) method was used for the gene expression calculation (Trapnell et al., 2010). Principal component analysis (PCA) was performed using the ggord package in R software. The differentially expressed genes (DEGs) were analyzed by DESeq (Anders et al., 2013) and defined as genes with |log_2_ fold change (FC)| ≥1, an adjusted *P*-value <0.05, a false discovery rate of <0.001. The GO and KEGG enrichment analyses of the DEGs were conducted to identify the enriched biological functions using the GOseq R software package (Young et al., 2010) and KOBAS software (Xie et al., 2011).

### Exogenous ACC treatment of rose leaves

Leaf stems were cut into 30 cm with three pairs of pinnate compound leaves and individually placed into 100 ml of 50 μM 1-aminocyclopropanecarboxylic acid (ACC) or deionized water as a control. After 24 h of treatment, the leaves were punched into discs.

### Ethylene and 1-MCP treatment ofrose leaves

Leaf stems placed in 100 ml deionized water were exposed to ethylene (10 μl/L), 1-methylcyclopropene (1-MCP, 2 μl/L), or regular air as a control for 24 h. 1M NaOH solution was added to the chambers to prevent CO_2_ accumulation. The leaves were then punched into discs for inoculation.

### Trypan blue staining assay

The rose leaves were harvested after inoculation for 0, 12, and 24 h and decolorized in the decolorizing solution (ethanol/glacial acetic acid = 1: 3) for 24–48 h until the leaves were bleached. The bleached leaves were stained with 0.05% trypan blue for 3 mins. The stained sections were visualized under an Olympus microscope. At least 4 leaves per time point were observed.

## Results

### RNA sequencing of rose seedling following *Podosphera pannosa* infection

In our previous study, after *P. pannosa* was infected for 12 h on rose leaves, the spores began to germinate and produce germinating tubes. Therefore, 12 hpi is the initiation of infection. At this time, the germination of spores produced a bud tube that extends into the epidermal cells of the rose leaves, and haustorium formed to obtain nutrients from plants, which is a key time point of initial infection. At 24 hpi, the conidia cluster formed, which means that the fungus was in the stage of asexual propagation. At this point, the second-generation spore was about to mature, and the second infection will occur soon. Accordingly, the third-generation spore will be produced and mature in the next 24 hours (**Supplementary Figure S1**).

**Figure 1:**
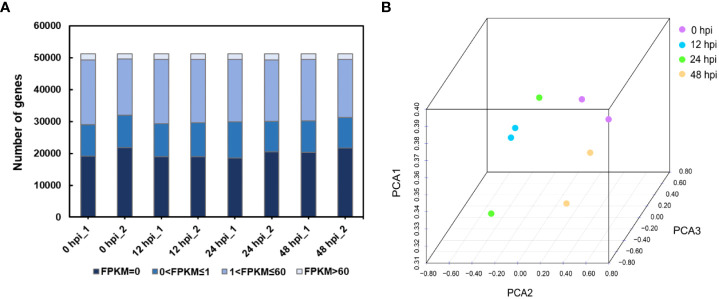
Overview of the RNA-seq data. **(A)** Distribution of the FPKM value in different samples. FPKM, fragments per kilobase per million reads. **(B)** Principal component analysis of rose RNA-seq data following *P. pannosa* infection.

Therefore, we chose the points of 0, 12, 24, and 48 hpi samples for transcriptome sequencing. The expression profiles were obtained from rose seedlings infected with *P. pannosa*. About 195 million clean reads from eight libraries were generated by 150-bp paired-end RNA sequencing. The quality of clean data was evaluated by FastQC. The results showed that the effective rate of each sample was more than 99%, with ~99% Q20, ~95% Q30, and ~46% GC content. The mapping rate of all samples was between 83.78 and 85.23% (**Supplementary Table S1**). The high quality of the clean data meets the transcriptome analysis requirements. As a result, 51,230 transcripts were identified in each sample. The gene expression level was evaluated by FPKM. The transcripts were classified by FPKM values, as shown in **Figure 1A**. There were about 19.8% of genes expressed at low levels (0 < FPKM ≤1), ~37.6% at moderate levels (1 < FPKM ≤60), and ~3.4 at high levels (FPKM >60). The FPKM of eight samples representing different infection time points was subjected to PCA. Although the two samples collected in 24 hpi were shown to be more discrete than the others, every two samples collected in one time point were able to cluster together, and the clear separation between different infection time points was detected (**Figure 1B**).

**Figure 2:**
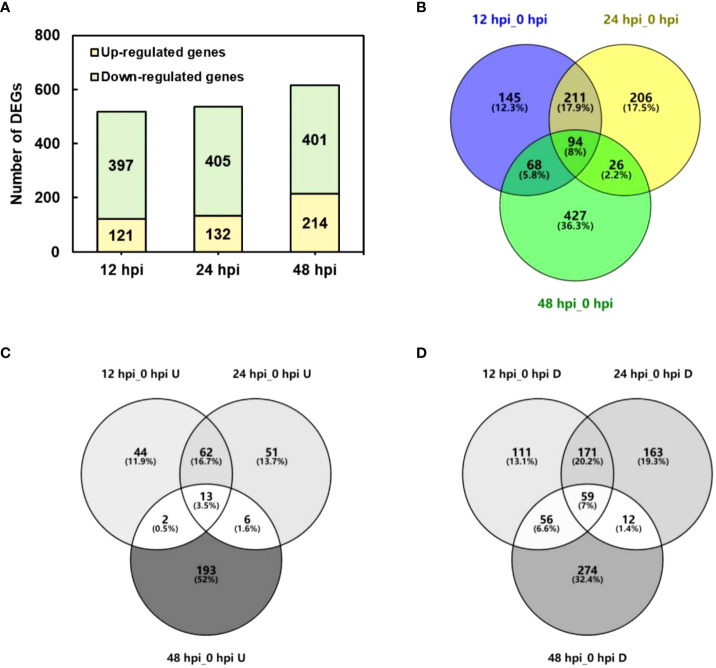
Number of DEGs in *P. pannosa*-infected rose seedlings at 12, 24, and 48 hpi. **(A)** Number of upregulated and downregulated DEGs at 12, 24, and 48 hpi. **(B–D)** Wayne figure of all upregulated and downregulated DEGs, respectively. DEGs, differentially expressed genes (cutoff ratio of >2, *p*-value <0.05, and *q*-value <0.05); U, upregulated DEGs; D, downregulated DEGs.

**Figure 3:**
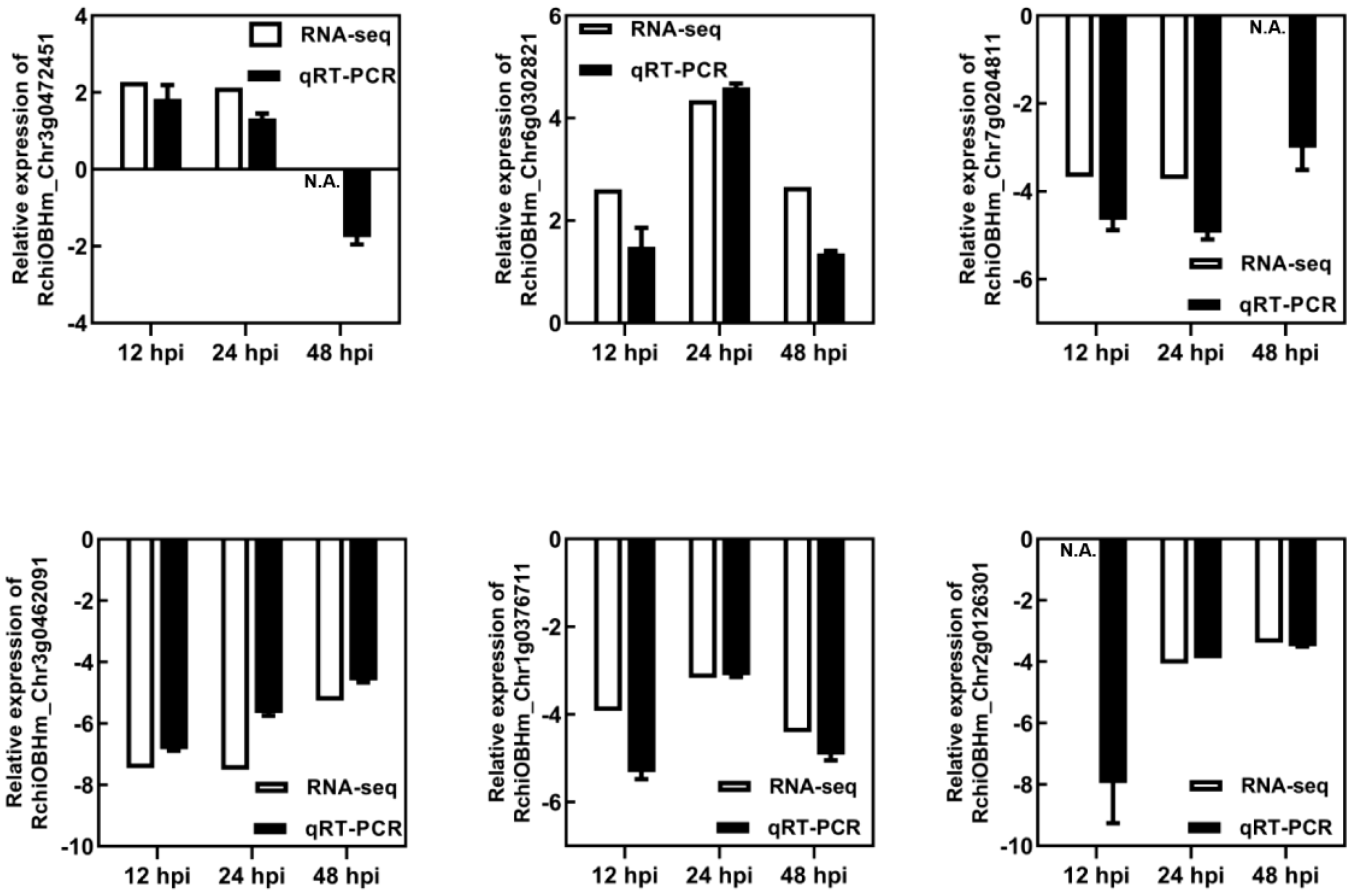
Validation of RNA-seq results using qRT-PCR. N.A. represented the RNA-seq data that was not available. RhUBI2 was used as the internal housekeeping gene control.

The DEGs were determined by DEGseq in comparison with 0 hpi. A total of 1,181 significant DEGs were obtained (the value of FPKM fold change was more than two times, *P*-value <0.05; **Figure 2**), of which 518, 537, and 615 DEGs were identified at 12, 24, and 48 hpi, respectively. Among the DEGs, 371 genes were upregulated once at least, and 846 genes were downregulated once at least in three points. In total, 94 DEGs showed a differential expression at all three time points, of which 13 were upregulated and 59 were downregulated. The results indicated that, during *P. pannosa* infection, the rose seedlings downregulated more genes than upregulated them, and the DEGs greatly varied in primary infection, re-infection, and tertiary infection. The qRT-PCR was used to validate the DEGs (**Figure 3**)

### The TFs involved in rose seedling defense response to *P. pannosa*

**Figure 4:**
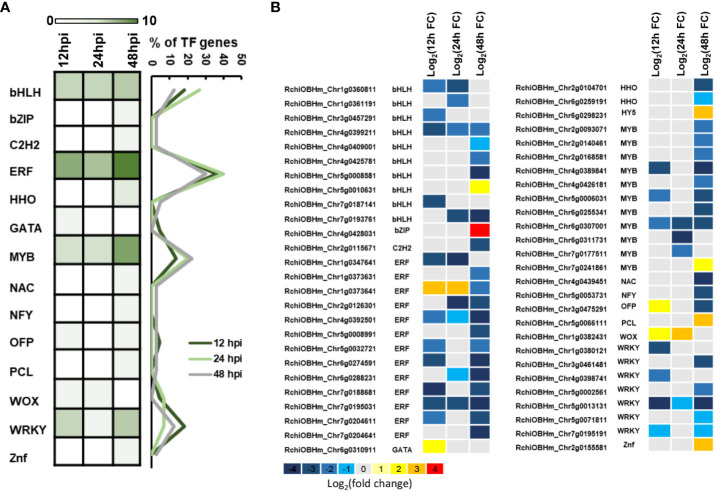
Kyoto Encyclopedia of Genes and Genomes (KEGG) pathways analysis for differentially expressed genes (DEGs). **(A)** KEGG pathway enrichment analysis of DEGs. **(B)** Relative expression changes of DEGs in plant hormone signal transduction pathway. ABA, abscisic acid; BR, brassinosteroid; ET, ethylene; GA, gibberellin; IAA, auxin; JA, jasmonic acid; SA, salicylic acid. The expression level was calculated using log_2_(FC). Log_2_(FC) = log_2_(fold change).

TFs are important components in plant defense, involving multiple cell physiological and molecular progress. Among 1,181 DEGs, we have identified 52 TFs involved in rose defense response once at least in three time points, including bHLH, bZIP, C2H2, ERF, HHO, GATA, MYB, NAC, NFY, OFP, PCL, WOX, WAKY, and Znf family members. The ERF family has the most of DEGs, followed by MYB, bHLH, and WRKY family, with 13, 11, 10, and seven DEGs, respectively (**Figure 4A**). There were 22, 15, and 40 TFs in 12, 24, and 48 hpi, respectively. Meanwhile, 80.2% of the TFs had a downregulated expression (**Figure 4B**). The downregulated TFs were sensitive to *P. pannosa*, and 10 of them were enriched in the KEGG analysis, which was related to the hormone signal transduction pathway and the MAPK signal pathway (**Supplementary Table S3**).

### Identified DEGs related to plant hormone signal transduction response to *P. pannosa* infection

**Figure 5:**
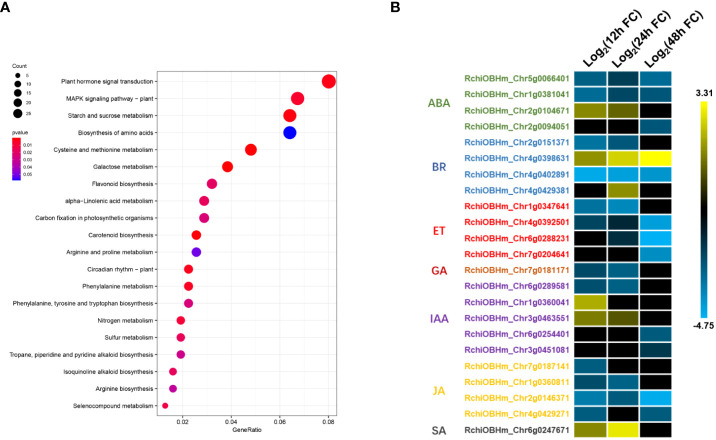
Differentially expressed transcription factors (TFs). **(A)** Number of TFs in *P. pannosa*-infected rose seedlings at 12, 24, and 48 hpi. **(B)** Relative expression changes of TFs at 12, 24, and 48 hpi. The expression level was calculated using log_2_(FC). Log_2_(FC) = log_2_(fold change).

To investigate the important biological progresses and pathways in rose seedlings resistant to *P. pannosa*, we annotated all the DEGs based on GO and KEGG databases. A total of 518, 516, and 461 DEGs were enriched with GO terms in biological progress, cellular component, and molecular function, respectively (**Supplementary Figure S2**). Interestingly, in the KEGG analysis, we have identified plant hormone signal transduction, MAPK signal pathway, starch and sucrose metabolism, *etc*., to be involved in rose response to *P. pannosa* (**Figure 5A**). The DEGs were significantly enriched in abscisic acid (ABA), brassinosteroid (BR), ET, gibberellins (GA), auxin (IAA), JA, and SA in the plant hormone signal transduction pathway. More importantly, the expression of DEGs in ET, JA, and GA was opposite to that in SA; they showed a consistently downregulated expression (**Figure 5B**). However, although the ABA-, BR-, and IAA-related DEGs were also enriched, their expression patterns at different time points were not consistent.

With the deepening of research on plant hormones, it is generally believed that SA is mainly the downstream signal of defense responding to the biotrophic pathogens, and JA often play opposite roles to SA in plant defense responses (Caarls et al., 2015). In our results, the JA- and SA-related DEGs also play an opposite expression pattern, and the results further suggested that ET may play a particular role in the rose–*P. pannosa* interaction.

### Exogenous ET inhibitor treatment improved rose resistance to *P. pannosa*

**Figure 6:**
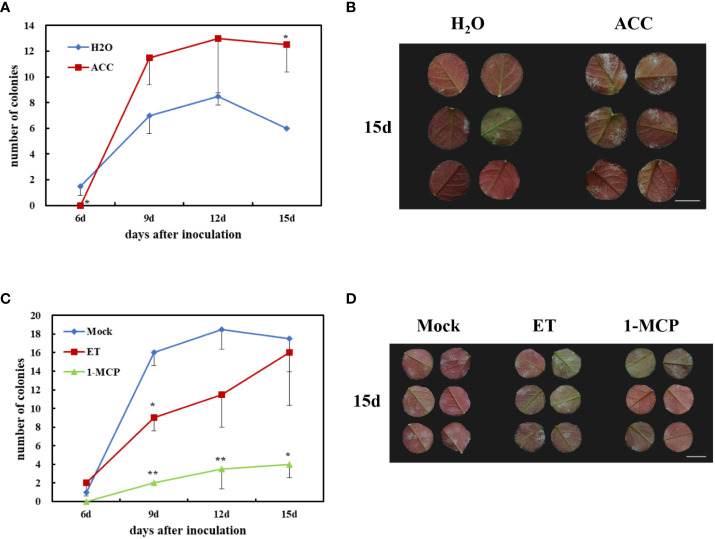
Effects of rose resistance to *P. pannosa* under exogenous 1-aminocyclopropanecarboxylic acid (ACC), ethylene (ET), and 1-methylcyclopropene (1-MCP) (ET inhibitor) treatments. **(A)** Quantification of *P. pannosa* colonies on rose leaves under ACC treatment. H_2_O was set as mock. Data statistics were carried out at 6, 9, 12, and 15 days after *P. pannosa* inoculation. **(B)** Typical appearance of control (H_2_O) and ACC-pre-treated rose seedlings at 15 days after *P. pannosa* inoculation. **(C)** Quantification of *P. pannosa* colonies on rose leaves under mock (air), ET, and 1-MCP. Data statistics were carried out at 6, 9, 12, and 15 days after *P. pannosa* inoculation. **(D)** Typical appearance of mock- (air), ET-, and 1-MCP-pre-treated rose seedlings at 15 days after *P. pannosa* inoculation. The asterisks indicate significant differences according to Student’s *t*-test (*P < 0.05, **P < 0.01).

ACC is an ethylene precursor, which is chemically stable and is often used as a substitute for gaseous ET to evaluate plant susceptibility to ethylene. To investigate the role of ET in rose defense response to *P. pannosa*, a 50-μM-ACC vase treatment of fresh rose leaf stem was carried out for 24 h. The variations of susceptibility to *P. pannosa* under ACC-pretreated leaves and mock (H_2_O-treated) leaves were observed at 6, 9, 12, and 15 dpi. The results showed that the ACC-treated rose leaves were more sensitive to *P. pannosa* than the mock leaves (**Figures 6A, B**). However, the gaseous-ET-pre-treated rose leaves showed no significant susceptibility to *P. pannosa*. On the contrary, the 1-MCP (the ET inhibitor)-pre-treated leaves showed a strong resistance to *P. pannosa* (**Figures 6C, D**). The results imply that 1-MCP can be used in the prevention of rose powdery mildew.

## Discussion

PM, caused by *P. pannosa*, is one of the most common and important fungal diseases of ornamental and horticultural plants around the world. *P. pannosa* is a biotrophic fungi which can be difficult to cultivate even though it is further studied compared with necrotrophic fungi. In agronomy cultivation, powdery mildew can infect the young leaves of roses, apples, strawberries, and other *Rosaceae* plants, which can lead to huge economic losses (Debener and Byrne, 2014; Vielba-Fernández et al., 2020). It is hence quite important and meaningful to study and explore the procedure and the mechanism behind *Pp.* infection on *Rosaceae* plants’ leaves. Here the rose cultivar ‘Samantha’ and *P. pannosa* (Pp) strains were used as materials to conduct transcriptome sequencing, and this highlighted massive genes and several hormone signal transduction pathways relevant to the resistance to rose powdery mildew.

In recent years, RNA-seq has been applied on several studies of plant–PM interaction, revealing the core DEGs and the biological progress in plant defense response. In this study, we have identified 1,181 DEGs once at least following three *Pp.* infection time points. Among the 1,181 DEGs, there were 846 downregulated genes and 371 upregulated genes. Consistently, in apple, jointly belonging to *Rosaceae*, 1,177 DEGs were identified between apple leaves subjected to fungal infection and those grown without pathogens at 12, 24, and 48 hpi (Tian et al., 2019). In pumpkin leaves, they obtained more than 3,000 DEGs after PM was inoculated for 24 and 48 h (Guo et al., 2018).

Transcriptional regulation is a central step in plant defense responses. Therefore, for understanding the molecular basis of plant–pathogen interactions, it is critical to elucidate the complex regulatory mechanisms that control defense gene expression among plant species. Here we have found 52 TFs with significantly differential expression, including four major family members (ERF, MYB, bHLH, and WRKY) and other 10 family members (**Figure 4**). Previous studies have shown that WRKY family members are involved in the regulation of plant disease resistance as well as in the regulation of transcriptional reprograming associated with plant immune responses (Eulgem and Somssich, 2007; Buscaill and Rivas, 2014). However, we identified ERF members as the most common in the TF family, similar to our previous study of rose petal defense response to *Botrytis cinerea* (Liu et al., 2018). The results indicated that ERF family members played an important role in rose defense response to both biotrophic and necrotrophic fungus. In recent years, there are more and more studies that show that ERF family members acted as a positive regulator in plant resistance to PM (Xing et al., 2017; Li et al., 2021; Zhang et al., 2021) and to grey mold (Pré et al., 2008; Catinot et al., 2015; Li et al., 2020).

The KEGG analysis demonstrated that most of the DEGs were enriched in plant hormone signal transduction pathway, including ABA-, AUX-, BR-, ET-, GA-, JA-, and SA-related genes. Although only one DEG was identified in the SA signal pathway, the expression pattern of this gene (RchiOBHm_Chr6g0247671) was opposite to that of the DEGs (RchiOBHm_Chr7g0187141, RchiOBHm_Chr1g0360811, RchiOBHm_Chr2g0146371, and RchiOBHm_Chr4g0429271) related to the JA pathway (**Figure 5**). Various research have pointed out that SA and JA form the basic bone of plant immunity system (De Vleesschauwer et al., 2013). It is generally believed that SA is thought to mediate defense signaling in response to biotrophic and hemibiotrophic pathogens, while JA and ET are associated with defense responses to necrotrophs, and these two pathways work in an antagonistic manner (Caarls et al., 2015). In addition, SA can inhibit a series of JA response genes (such as PDH1.2) and JA biosynthesis-related genes (LOX2/AOS/AOC2/OPR3) (Leon-Reyes et al., 2010).

In this study, four DEGs were found in the ET signaling pathway. Interestingly, all of the four DEGs were downregulated during *Pp.* infection (**Figure 5**). These results indicated that ET may play a negative role in rose defense response to *Pp.* Ethylene is a gaseous plant hormone, which is involved in regulating the physiological and biochemical processes of many plants, including seed germination, plant growth, fruit ripening, organ abscission, and aging (Schulzelefert and Vogel, 2000). In addition, when plants are attacked by pathogens and herbivores, ethylene also plays an important role in plant defense system (Broekaert et al., 2006; Broekgaarden et al., 2015). ACC is the premise of ethylene synthesis to replace ethylene and added hormones to water agar to ensure the continuous supply of ethylene. The results showed that the treatment group had a later onset than the control group, had a significant difference at the time of inoculation for 6 days, then exceeded the control group (H_2_O), and reached a significant difference at the time of inoculation for 15 days (**Figures 6A, B**). While we first found that 1-MCP, a gas ethylene inhibitor, can significantly improve the disease resistance of rose leaves after 9 dpi, gas ET treatment showed increased resistance at 9 dpi and gradually approached the control group at 15 dpi (**Figures 6C, D**). We speculated that the different phenotype between ACC and gas ET treatment may be due to the fact that the gas ethylene treatment in a short day cannot play a role in the detached leaves continuously. The continuous energy supply of ACC keeps producing gas ethylene, which makes the ethylene concentration in the inoculated environment higher and higher. Therefore, based on the above-mentioned results, we clarified that 1-MCP could be a candidate for exogenous chemical agents for the economic and sustainable improvement of rose resistance to PM.

## Conclusions

In conclusion, this study focused on rose early defense response to powdery mildew and identified 1,181 DEGs during infection. In total, 52 differentially expressed TFs, especially ERF, MYB, bHLH, and WRKY family members, consisted of the core regulatory factor network. Simultaneously, besides SA and JA, the KEGG enrichment highlighted the ET signaling pathway in the regulation of rose leaves’ resistance. The application test of 1-MCP, an ethylene inhibitor, showed that 1-MCP played a positive role in rose resistance to PM.

## Data availability statement

The datasets presented in this study can be found in online repositories. The names of the repository/repositories and accession number(s) can be found below: https://www.ncbi.nlm.nih.gov/, PRJNA661227.

## Author contributions

XL and PF performed the experiments. XL, PF, XiaC, and ZW analyzed the data. XL, XQC, and ZZ complemented the writing of the manuscript. XiC and ZZ planned the project, designed and supervised the experiments, and coordinated the collaboration of the authors. All authors contributed to the article and approved the submitted version.

## Funding

This work was supported by the National Natural Science Foundation of China (grant number 31501791), and the Construction of Beijing Science and Technology Innovation and Service Capacity in Top Subjects (CEFF-PXM2019_ 014207_000032) to ZZ. This study was further sponsored by the Natural Science Foundation of Jiangsu Province (BK20191224) and supported by the project of Jiangsu Vocational College of Agriculture and Forest (2019kj005) to XC, also the China Postdoctoral Science Foundation (2022M713391) to XL.

## Conflict of interest

The authors declare that the research was conducted in the absence of any commercial or financial relationships that could be construed as a potential conflict of interest.

## Publisher’s note

All claims expressed in this article are solely those of the authors and do not necessarily represent those of their affiliated organizations, or those of the publisher, the editors and the reviewers. Any product that may be evaluated in this article, or claim that may be made by its manufacturer, is not guaranteed or endorsed by the publisher.
