# Supplementary material for: Comparative RNA-seq analysis reveals a critical role for ethylene in rose (Rosa hybrida) susceptible response to Podosphera pannosa
Source: Front Plant Sci. 2022 Sep 27;13:1018427. doi: 10.3389/fpls.2022.1018427 (PMC9551381; doi:10.3389/fpls.2022.1018427)
Supplement: Supplementary file 1 [file DataSheet_1.docx]

**The list of f Supplementary data**

Supplementary Figure 1. Microscopic observation of powdery mildew fungal structure on rose leaves.

Supplementary Figure 2. GO analysis for DEGs.

Supplementary Table 1. Quality Summary of clean data.

Supplementary Table 2 List of Abbreviations.

Supplementary Table 3 The DEGs in *P. pannosa* infected rose seedlings at 12 hpi, 24hpi and 48 hpi.

**Supplementary Figures and Tables**

**
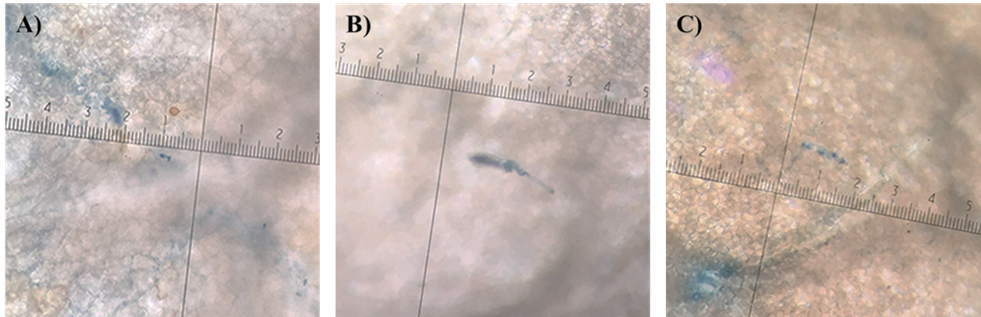
**

**Supplementary Figure 1. Microscopic observation of powdery mildew fungal structure on rose leaves.** Inoculated by *P. pannosa* at 0 h (A), 12h (B) and 24h (C)

**
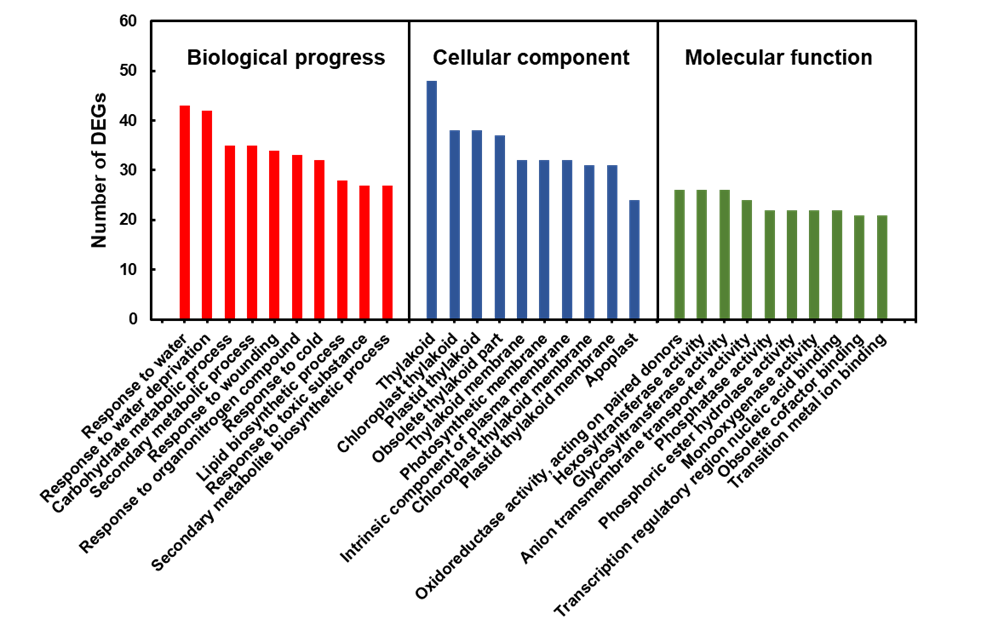
**

**Supplementary Figure 2. GO analysis for DEGs.** The top ten components in biological progress, cellular component and molecular function were showed on this figure.

**Supplementary Table 1. Quality Summary of clean data**

| **Sample_**  **name** | **Clean_base** | **Clean reads** | **Effective rate（%）** | **Q20** | **Q30** | **GC content（%）** | **Mapping to *R. chinensis*（%）** |
| --- | --- | --- | --- | --- | --- | --- | --- |
| 0 h_1 | 3444421200 | 22962808 | 99.13% | 99.83% | 95.70% | 46.10 | 84.18% |
| 0 h_2 | 3760925400 | 25072836 | 98.57% | 99.83% | 95.71% | 46.09 | 83.78% |
| 12 h_1 | 3512015400 | 23413436 | 99.22% | 99.81% | 95.50% | 45.78 | 85.23% |
| 12 h_2 | 4258662300 | 28391082 | 98.14% | 99.85% | 95.86% | 46.02 | 84.66% |
| 24 h_1 | 4858910100 | 32392734 | 98.51% | 99.82% | 95.53% | 45.81 | 84.91% |
| 24 h_2 | 2961297000 | 19741980 | 98.44% | 99.70% | 94.21% | 45.75 | 84.07% |
| 48 h_1 | 3383909850 | 22559399 | 98.78% | 99.73% | 94.55% | 46.22 | 84.47% |
| 48 h_2 | 3033625950 | 20224173 | 98.01% | 99.71% | 94.36% | 45.89 | 84.15% |

All data were counted for read1+read2. Clean bases: (Clean reads)×(sequence length). Effective Rate (%): (Clean reads/Raw reads)×100%. Error rate: base error rate Q20, Q30: (Base count value > 20 or 30) / (Total base count). GC content: (G & C base count) / (Total base count).

**Supplementary Table 2 List of Abbreviations**

| **Abbreviations** | **Full name** |
| --- | --- |
| 1-MCP | 1-methylcyclopropene |
| ABA | abscisic acid |
| ACC | 1-Aminocyclopropanecarboxylic Acid |
| Aux | Auxin |
| BR | brassionosteroid |
| DEG | differentially expressed genes |
| DPI | days post inoculation |
| ET | ethylene |
| FC | fold change |
| FPKM | fragments per kb per million reads |
| GA | Gibberellin |
| GO | gene ontology |
| HPI | Hours post inoculation |
| JA | jasmonic acid |
| KEGG | kyoto encyclopedia of genes and genomes enrichment |
| NR | non-redundant protein |
| NT | NCBI non-redundant transcript |
| PCA | principal component analysis |
| PM | powdery mildew |
| SA | salicylic acid |
| TF | transcription factor |
